# Guillain-Barré Syndrome with Facial Diplegia Related to SARS-CoV-2 Infection

**DOI:** 10.1017/cjn.2020.106

**Published:** 2020-05-29

**Authors:** Jason L. Chan, Hamid Ebadi, Justyna R. Sarna

**Affiliations:** Department of Clinical Neurosciences, University of Calgary, Calgary, Alberta, Canada T2N 1N4; Hotchkiss Brain Institute, University of Calgary, Calgary, Alberta, Canada T2N 4N1

**Keywords:** Acute inflammatory demyelinating polyneuropathy, COVID-19, Cranial neuropathy, Peripheral nervous system, Polyradiculoneuropathy

Severe acute respiratory syndrome coronavirus 2 (SARS-CoV-2) was first detected in December 2019 and is the cause of the ongoing worldwide pandemic of coronavirus disease 2019 (COVID-19). Patients with COVID-19 commonly present with fever, fatigue, cough, and shortness of breath. However, neurological manifestations of COVID-19 are becoming increasingly recognized and include impairment of smell and taste, acute cerebrovascular disease, and encephalopathy.^1^

Guillain-Barré syndrome (GBS) is the most common cause of weakness due to acute polyradiculoneuropathy and consists of immune-mediated demyelinating and axonal forms.^2^ It is typically post-infectious, with onset occurring within 4 weeks of symptoms of respiratory tract or gastrointestinal infection in two-thirds of patients.^2^ Cases of GBS associated with COVID-19 have been rarely but increasingly reported.^3–12^

A 58-year-old, right-hand-dominant male who was otherwise healthy presented with acute-onset bilateral facial weakness, dysarthria, and paresthesia in his feet. He denied any other neurological symptoms, including anosmia and ageusia. He denied fever, fatigue, cough, shortness of breath, or any other symptoms on review of systems. Notably, the patient and an immediate family member work at a meat-processing plant and were exposed to a known workplace outbreak of SARS-CoV-2. Subsequently, the patient was physical distancing and isolating at home in the 20 days prior to his presentation.

Initial vital signs included a temperature of 36.6 °C, maximum heart rate of 140 beats/minute, maximum blood pressure of 187/103 mmHg, maximum respiratory rate of 34 breaths/minute, and an oxygen saturation of 96% on room air, with resolution of tachycardia, hypertension, and tachypnea within 12 hours. Auscultation of the lungs revealed diffuse crackles bilaterally. Neurological examination demonstrated complete facial diplegia and areflexia in the lower extremities. He could not raise his eyebrows and there was no creasing of the forehead. With attempted eye closure, the palpebral fissures remained symmetrically open with a width of 5 mm. His nasolabial folds were flattened symmetrically, and he could not smile or close his lips. He had dysarthria with labial sounds. The remainder of the neurological examination was normal. In particular, he had full strength, normal sensation to pinprick, temperature, vibration, and proprioception, and absence of ataxia in his upper and lower extremities.

**Figure 1:**
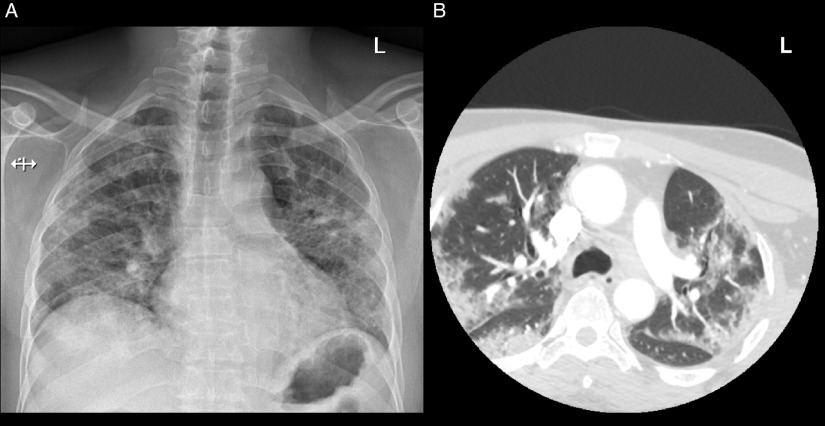
Lung imaging. (A) Chest x-ray demonstrated diffuse heterogeneous infiltration in both lungs and corresponded with (B) CTA of the head and neck, which demonstrated ground-glass opacities in the lung apices, involving primarily the periphery in the upper lobes of the lungs and the superior segments of the lower lobes. These findings were consistent with COVID-19. L = left.

**Figure 2:**
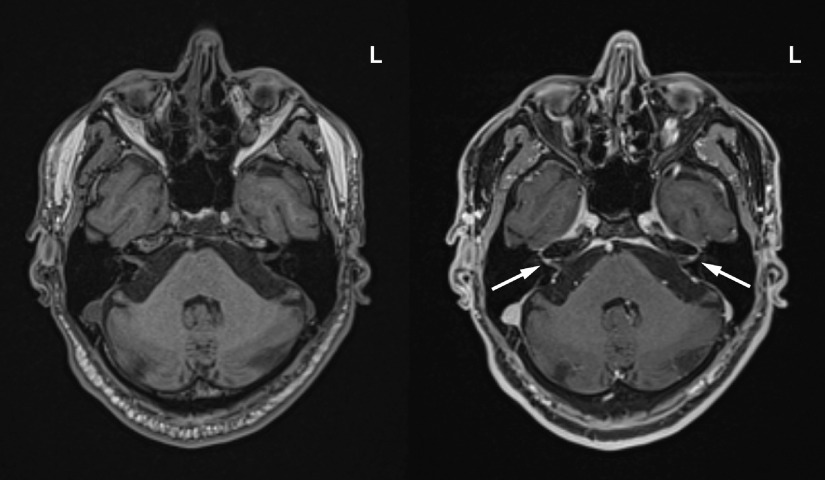
Axial T1-weighted pre-contrast (left) and T1-weighted fat-saturated post-contrast brain MRI (right) demonstrated bilateral facial nerve enhancement involving the labyrinthine segment (arrows), tympanic segment, mastoid segment, and extracranial facial nerve. L = left.

Laboratory investigations demonstrated a persistent thrombocytosis with a maximum platelet count of 688 × 10^9^/L (normal 150–400 × 10^9^/L) and an elevated D-dimer at 1.47 mg/L (normal ≤ 0.5 mg/L). Oropharyngeal RT-PCR testing for SARS-CoV-2 was positive and chest X-ray demonstrated diffuse heterogeneous infiltration in both lungs (Figure 1 A). Computed tomography (CT) and CT angiography (CTA) of the head and neck did not demonstrate any intracranial or vascular abnormalities but demonstrated ground-glass opacities in both lung apices, consistent with COVID-19 (Figure 1B). The patient was immediately started on empiric ceftriaxone and azithromycin, given his risk of developing pneumonia. Bilateral intracranial and extracranial facial nerve enhancement was present on brain magnetic resonance imaging (MRI) (Figure 2). Cerebrospinal fluid (CSF) analysis demonstrated cytoalbuminologic dissociation with a protein of 1.00 g/L (normal 0.15–0.45 g/L) and a white blood cell count of 4 × 10^6^/L (normal ≤ 5 × 10^6^/L). CSF RT-PCR testing for SARS-CoV-2 was negative.

Overall, the patient’s history, examination, and investigations were consistent with GBS. Given the possibility of hypercoagulability associated with COVID-19 and the patient’s persistent thrombocytosis, the safety profiles of intravenous immunoglobulin (IVIG) and plasma exchange were discussed with colleagues in hematology. The thrombosis risks were thought to be equivalent and 2 days after symptom onset, the patient started a 5-day course of IVIG at 0.4 g/kg/day. Electrophysiological studies were performed 6 days after symptom onset and demonstrated absent blink reflexes bilaterally and an absent F wave in the left tibial nerve, consistent with acute inflammatory demyelinating polyneuropathy. Anti-ganglioside antibodies were not tested. The patient was discharged from hospital 2 days after completing IVIG. At that time, he had slight movements of his facial muscles, and the distal paresthesias of his lower extremities were unchanged.

Interestingly, GBS was the presenting disorder that led to a diagnosis of COVID-19. Although the patient was asymptomatic from a respiratory perspective, his positive SARS-CoV-2 test and lung imaging (Figure 1) were indicative of an active infection at the time of GBS onset. He had a clear source of infection from an outbreak and his subsequent isolation at home for 20 days further suggested that his infection preceded GBS onset. Taken together, COVID-19 may manifest primarily with neurological symptoms and signs. In context of the current pandemic, a patient with GBS should prompt consideration for a preceding or concurrent SARS-CoV-2 infection. Furthermore, assessment of acute neurological presentations with CTA of the head and neck can assist in diagnosing COVID-19 based on simultaneous CT imaging of the lung apices.

At the time of writing, at least 14 cases of GBS associated with COVID-19 have been reported worldwide: 12 cases presented with symmetric, progressive, ascending weakness,^3-11^ 1 case with facial diplegia,^5^ and 1 case with Miller Fisher syndrome.^12^ In the 13 cases where COVID-19 symptoms preceded GBS symptoms, the interval ranged from 5 to 24 days.^4-12^ Of the 12 cases with electrophysiological studies, 4 had an axonal process^4,5^ and 8 had a demyelinating process.^3,5,7–11^ CSF RT-PCR testing for SARS-CoV-2 was negative in 8 cases.^5,8,11,12^ All 14 cases were treated with IVIG, with 2 cases receiving a second cycle of IVIG and 1 case receiving subsequent plasma exchange.^3–12^

Typically, infections such as *Campylobacter jejuni*, cytomegalovirus, Epstein–Barr virus, influenza A virus, *Mycoplasma pneumoniae*, *Haemophilus influenzae*, and Zika virus are thought to cause GBS through an autoimmune reaction via molecular mimicry.^2^ The mechanisms of GBS associated with COVID-19 are unclear. Neuroinvasive potential is a common feature of other human coronaviruses (HCoVs), such as SARS-CoV, Middle East respiratory syndrome coronavirus (MERS-CoV), HCoV-229 E, and HCoV-OC43,^13^ and has led to the hypothesis that SARS-CoV-2 may act directly on the nervous system to cause neurological symptoms. The absence of SARS-CoV-2 in the CSF in this case and others^5,8,11,12^ does not support this hypothesis with regard to GBS.

COVID-19 is associated with increased levels of proinflammatory cytokines and systemic inflammatory response syndrome,^14^ which favor an inflammatory or immunological mechanism. As such, cytoalbuminologic dissociation, similar to typical GBS, may be a result of antibody production via molecular mimicry, increased release of myelin protein from axons, or blood–nerve barrier dysfunction. GBS associated with COVID-19 may involve post-infectious or para-infectious processes, as cases have had preceding or concurrent SARS-CoV-2 infections and symptoms, respectively. Future investigation will be required to elucidate the mechanisms of GBS associated with COVID-19.

The incidence of GBS is approximately 1–2 cases per 100,000 people per year and changes in incidence may correspond with exposure to infectious agents known to cause GBS.^2^ For example, during the 2015–2016 Zika virus epidemic, increases and decreases in GBS incidence worldwide coincided with increases and decreases in Zika virus disease incidence, with increases in GBS incidence varying between 2 to 10 times the baseline.^15^ Nonetheless, GBS associated with Zika virus remained an uncommon disorder. Although there is increasing evidence that GBS may occur secondary to COVID-19 and cases may be underreported, the rate is likely consistent with that associated with other infectious diseases. As the COVID-19 pandemic continues, GBS and other neurological manifestations of COVID-19 will continue to be characterized.
